# Paternal factors and adverse birth outcomes in Lanzhou, China

**DOI:** 10.1186/s12884-020-03492-9

**Published:** 2021-01-06

**Authors:** Jing Li, Jie Qiu, Ling Lv, Baohong Mao, Lei Huang, Tao Yang, Cheng Wang, Qing Liu

**Affiliations:** 1 Research center, Gansu Provincial Maternity and Child Care Hospital, Lanzhou, Gansu China; 2 Obstetrical department, Gansu Provincial Maternity and Child Care Hospital, Lanzhou, Gansu China; 3 Pediatrics department, Gansu Provincial Maternity and Child Care Hospital, Lanzhou, Gansu China; 4 Galactophore department, Gansu Provincial Maternity and Child Care Hospital, Lanzhou, Gansu China; 5 Gynaecology department, Gansu Provincial Maternity and Child Care Hospital, Lanzhou, Gansu China

**Keywords:** Low birth weight, Preterm birth, Small for gestational age, Birth outcome, Paternal

## Abstract

**Background:**

Many maternal factors are known to be associated with adverse birth outcomes, but studies about paternal factors yielded inconsistent conclusions. The study was to assess whether paternal factors are associated with low birth weight (LBW), preterm birth (PTB), and small for gestational age (SGA).

**Methods:**

A birth cohort study was conducted in 2010–2012 at the Gansu Provincial Maternity and Child Care Hospital, the largest maternity and childcare hospital in Lanzhou, China. Paternal age, ethnicity, educational level, height, weight, smoking, and drinking were collected. Birth outcomes and pregnancy complications were extracted from the medical records.

**Results:**

During the study period, 10,121 participants were included; the overall prevalence of LBW, PTB, and SGA was 7.2, 9.9, and 7.8%, respectively. Paternal higher height (OR = 0.64 95%CI: 0.49, 0.83), higher weight (P for trend < 0.001), and higher BMI (P for trend < 0.001) could decrease the rate of LBW. Paternal higher education (OR = 0.55, 95%CI: 0.43, 0.71) and higher weight (P for trend < 0.001,) were associated with lower rate of PTB. Fathers who smoked more than 6 pack-years were associated with PTB (OR = 1.31, 95%CI: 1.07, 1.61). Paternal BMI > 23.9 kg/m^2^ (P for trend < 0.001,) and paternal education which above college (OR = 0.61, 95%CI: 0.50, 0.82) were associated with a lower rate of SGA.

**Conclusion:**

Paternal low education is independently associated with PTB and SGA. Paternal heavy smoking is associated with PTB. Low paternal weight/BMI is independently associated with LBW, PTB, and SGA.

**Supplementary Information:**

The online version contains supplementary material available at 10.1186/s12884-020-03492-9.

## Background

Various adverse birth outcomes including low birth weight (LBW), preterm birth (PTB), and small for gestational age (SGA) are associated with increased neonatal morbidity and mortality [1], and even have a long-term impact in childhood and adulthood [2]. Many maternal factors have been proved to be related to adverse birth outcomes, including advanced maternal age, passive smoking, and hypertensive disorders during pregnancy, but few studies paid attention to the influence of paternal factors on their offspring.

Studies that investigated the relationship between paternal factors and adverse birth outcomes have reached inconsistent results. Some studies observed that advanced paternal age was associated with an increased risk of LBW [3] and PTB [4], while others showed that young paternal age was a risk factor for LBW, PTB, and SGA [4, 5]. Several studies reported no associations between paternal age and LBW [6], PTB [7], and SGA [6]. Socioeconomic status (SES), especially education, has been suggested to be associated with different adverse birth outcomes, but paternal SES information is not routinely collected in most studies. Four studies reported that less than a high school education was associated with higher rates of PTB [8] and SGA [9, 10]. Another study found that education less than the college level was associated with higher odds of LBW births [11]. Regarding paternal anthropometry, paternal height was found to have an independent effect on birth weight in the shortest fathers having offspring with lower birth weight compared with the tallest fathers [6, 12]. On the other hand, some studies showed no significant relationship between paternal anthropometry and birth outcomes [6]. In addition, several studies indicated that paternal smoking had significant impact on their offspring’s birth weight, which was 30–130 g lower [13]. Recently, Ko et al. [14], Inoue et al. [15], and Fan et al. [16] showed that paternal smoking did not have any significant relationship with LBW, PTB, and SGA. Lastly, Little & Sing [17] showed a decrease in birth weight (of 180 g) associated paternal alcohol consumption, whereas several epidemiological studies report no relationship between paternal alcohol consumption and birth weight [16].

Compared with Beijing, Shanghai and Guangzhou, Lanzhou is an economically underdeveloped area located in Northwest China. At present, no study has assessed the perinatal outcomes in Northwest China using a large birth cohort. Therefore, the aim of this study was to assess whether paternal factors are associated with LBW, PTB, and SGA.

## Methods

### Study design and subjects

A birth cohort study was conducted in 2010–2012 at the Gansu Provincial Maternity and Child Care Hospital, the largest maternity and childcare hospital in Lanzhou, China [18]. The study protocol was approved by the human investigation committees at the Gansu Provincial Maternity and Child Care Hospital and Yale University. All patients provided informed written consent. Pregnant women who came to the hospital for delivery at 20 weeks of gestation or more, who had no mental illness, and who were at least 18 years of age were eligible.

### Data collection

Upon obtaining written consent, five residents trained in epidemiology at the Yale School of Public Health conducted in-person interviews at the hospital using a standardized and structured questionnaire (Supplementary File 1). The women were interviewed within 1 to 3 days after delivery. The questionnaire included reproductive and medical histories, smoking, alcohol and tea consumption, occupational and residential histories, physical activity, and diet. Information on birth outcomes and pregnancy complications were extracted from the medical records.

Information on paternal characteristics was also collected through the questionnaire and confirmed by the fathers, including age, height, weight, reproductive and medical histories, smoking, alcohol consumption, occupational and residential histories were collected within 1 to 3 days after delivery. Paternal age was defined as the age of father in completed years at the time of their child’s birth. Paternal body mass index (BMI) (kg/m^2^) was calculated from paternal weight and height data were measured by nurses after the newborns were delivered, with the fathers paying a visit to the mothers. Smokers were defined as men who smoked 1 or more cigarettes per day for at least 1 month before deliveries. Drinker was defined as men who were exposed to alcohol at least one time per week on average before deliveries. Based on tertiles of, height, and weight, packyear, drinking times per year were classified into three groups. The first tertiles were used as reference groups.

### Birth outcomes

LBW was defined as birth weight < 2500 g, regardless of gestational age. The gestational age at delivery was calculated in completed weeks from the first day of the last menstrual period. PTB was defined as the birth with gestational age < 37 completed weeks [19]. SGA birth was defined as an infant born with a birth weight below the 10th percentile of the gestational age- and gender-specific birth weight standards for Chinese newborns [20]. Neonates who weighed between the 10th and 90th percentiles were defined as appropriate for gestational age (AGA) births. The range of Chinese national standard is 28–44 weeks. For neonates with a gestational age of 22–27 weeks, the US national reference based on the 2009–2010 US live births was used as a surrogate standard [21]. Since no gestational age- and gender-specific birth weight standards are available for gestational age < 22 weeks, four participants with gestational age less than 22 weeks were excluded from the analysis for SGA.

### Statistical analysis

All data were managed using EpiData 3.0 (Centers for Disease Control, Atlanta, GA, USA). The continuous data were tested for normal distribution using the Kolmogorov-Smirnov test. Continuous data are presented as mean ± standard deviation and analyzed using the Student t-test or ANOVA with the Tukey’s post hoc test (normal distribution), or as median (range) and the Mann-Whitney U test (non-normal distribution). Categorical variables are presented as frequencies and were analyzed using the Fisher’s exact test. Multiple logistic regression models were used to estimate odds ratios (OR) and 95% confidence intervals (95%CI) for the associations between paternal characteristics and birth outcomes. Based on the literature [1, 15], the following maternal variables were included in the final models as potential confounders: maternal age (< 25, 25–29, 30–34, and ≥ 35 years), educational attainment (less than high school graduation, high school and community college, and higher than college), employment status during pregnancy (yes or no), parity (nulliparous or parous), maternal pre-pregnancy BMI (< 18.5, 18.5–23.9, > 23.9 kg/m^2^), hypertensive disorders during pregnancy (yes or no), family’s average monthly income (≤3000, > 3000 yuan/month), smoking during pregnancy (yes or no), and gestational week (only for LBW). Adjustment for cesarean delivery of the current birth (yes or no), maternal gestational diabetes (yes or no), maternal alcohol consumption (yes or no), and infant gender (male or female) did not result in changes in the observed associations and thus were not included in the final models. Two-sided *P*-values < 0.05 were considered statistically significant. All statistical analyses were carried out using SAS 9.4 (SAS Institute, Cary, NC, USA).

## Results

### Subjects

A total of 14,535 pregnant women came to the hospital for delivery, of whom 176 were judged to be ineligible for the study (13 had mental illness, 39 were younger than 18 years of age, and 124 gave birth at less than 20 gestational weeks). Thus, a total of 14,359 eligible women were approached for participation. Of those, 3712 refused to participate and 105 did not complete in-person interviews, which yielded 10,542 (73.4%) women who completed in-person interviews. After exclusion of women who had multiple births (*n* = 323), stillbirth (*n* = 53), and missing information on infant gender (*n* = 36) and birth weight (*n* = 43), and missing information on father’s factors (*n* = 34) or women with more than one exclusion conditions, 10,121 participants were included in the analysis.

### Maternal and paternal characteristics

The selected parental baseline characteristics are shown in Table 1. The mean age of the mothers and fathers was 28.6 ± 4.4 years and 30.7 ± 4.9 years, respectively. Before pregnancy, 19.9 and 3.1% of women and men were underweight. The literacy rate was high: 37.2% in women and 40.8% in men. About one in five mothers (19.6%) were smoking, either actively or passively, and this situation was more common in men (48.3%). Drinking was observed in 0.2 and 22.6% of the mothers and fathers, respectively. Most families had low incomes (< 3000 yuan/month) (50.5%).

**Table 1:** Parental baseline characteristics of the total study population (*n* = 10,121), Urban China, 2010–2012

Characteristics	Mother (*n* = 10,121)	Father (*n* = 10,121)
Age (years) (mean ± SD)	28.6 ± 4.4	30.7 ± 4.9
< 25 years	1624 (16.1%)	804 (7.9%)
25 to < 30 years	4830 (47.7%)	3541 (35.0%)
30 to < 35 years	2701 (26.7%)	3821 (37.8%)
≥ 35 years	966 (9.5%)	1955 (19.3%)
Ethnic group		
Han	9415 (93.0%)	9291 (91.8%)
Others	706 (7.0%)	830 (8.2%)
BMI (kg/m^2^) (mean ± SD)	20.68 ± 2.73	23.83 ± 3.08
< 18.5	2013 (19.9%)	317 (3.1%)
18.5–23.9	6682 (66.0%)	5084 (50.2%)
≥ 24	1079 (10.7%)	4720 (46.6%)
Missing	347 (3.4%)	0
Education status		
Less than high school graduation	2225 (22.0%)	2004 (19.8%)
high school and community college	3950 (39.0%)	3990 (39.4%)
higher than college	3761 (37.2%)	4127 (40.8%)
Missing	185 (1.8%)	0
Smoking ^a^		
No smoking	8136 (80.4%)	5232 (51.7%)
Smoking	1985 (19.6%)	4889 (48.3%)
Alcohol use		
No	10,101 (99.8%)	7827 (77.3%)
Yes	20 (0.2%)	2294 (22.6%)
Employment status		
No	4897 (48.4%)	1086 (10.7%)
Yes	5224 (51.6%)	9035 (89.3%)
Sex of the child		
boy	5343 (52.8%)	
girl	4778 (47.2%)	
Parity		
Nulliparous	7307 (72.2%)	
Parous	2814 (27.8%)	
Cesarean delivery		
No	6179 (61.1%)	
Yes	3850 (38.0%)	
Missing	92 (0.9%)	
Hypertensive disorders during pregnancy		
No	9600 (94.9%)	
Yes	521 (5.2%)	
Diabetes		
Yes	103 (1.0%)	
No	10,018 (99.0%)	
Family’s average monthly income (yuan)		
≤ 3000	5113 (50.5%)	
> 3000	4038 (39.9%)	
missing	970 (9.6%)	

^a^ Maternal smoking including active smoking and passive smoking

^b^ The analysis did not account for missing data

### Birth outcomes

The overall prevalence of LBW was 7.2%. As shown in Table 2, compared with the normal birth weight group, the ORs of LBW were 0.74 (95% CI: 0.56, 0.97) and 0.68 (95% CI: 0.52, 0.88) for fathers who had higher and the highest weight, respectively. When adding the maternal and paternal factors, paternal higher weight (OR = 0.75, 95% CI: 0.55, 1.02) and the highest weight (OR = 0.66, 95%CI: 0.43, 1.01) were not significantly associated with decreased risk of LBW. Compared with infants born with normal weight, the fathers whose BMI was > 24 kg/m^2^ were associated with a lower rate of LBW (OR = 0.56, 95%CI: 0.32, 0.97). Compared with the control group, young paternal age, small paternal stature, paternal smoking, and paternal drinking were not associated with LBW. Interesting, father who liked drinking beer and red wine was associated with LBW (OR = 5.47, 95%CI: 1.03, 29.18), although these findings were based on only 3 LBW cases and should be cautiously interpreted.

**Table 2:** Association between paternal factors and LBW by multiple logistic regression analysis

Paternal factors	Normal BW (***N*** = 8710) (***n***/%)	LBW (***N*** = 727) (***n***/%)	OR (95%CI)^**a**^	OR (95%CI)^**b**^
Age				
< 25	678 (7.8%)	94 (12.9%)	ref.	ref.
25 to < 30	3059 (35.1%)	239 (32.9%)	1.04 (0.68,1.61)	1.06 (0.68,1.64)
30 to < 35	3340 (38.4%)	233 (32.1%)	0.92 (0.58,1.47)	0.96 (0.59,1.53)
≥ 35	1633 (18.8%)	161 (22.2%)	0.92 (0.53,1.58)	0.93 (0.53,1.61)
*P* for trend			0.392	0.446
Ethnicity				
Han	8008 (91.9%)	648 (89.1%)	ref.	ref.
Others	702 (8.1%)	79 (10.9%)	0.73 (0.40,1.31)	0.71 (0.39,1.27)
Education years (%)				
Less than high school graduation	1610 (18.5%)	292 (40.2%)	ref.	ref.
high school and community college	3456 (39.7%)	257 (35.4%)	0.76 (0.55,1.05)	0.81 (0.59,1.12)
higher than college	3644 (41.8%)	178 (24.5%)	0.79 (0.53,1.20)	0.87 (0.53,1.33)
Height (m)^c^				
< 1.71	2663 (30.6%)	292 (40.2%)	ref.	ref.
1.71–1.74	2190 (25.1%)	188 (25.9%)	0.82 (0.62,1.09)	0.82 (0.62,1.09)
≥ 1.75	3857 (44.3%)	247 (34.0%)	**0.64 (0.49,0.84)**	**0.64 (0.49,0.83)**
*P* for trend			**0.002**	**0.002**
Weight (Kg)				
< 67.0	2744 (31.5%)	310 (42.6%)	ref.	ref.
67–74.9	2431 (27.9%)	188 (25.9%)	**0.74 (0.56,0.97)**	0.75 (0.55,1.02)
≥ 75	3535 (40.6%)	299 (31.5%)	**0.68 (0.52,0.88)**	0.66 (0.43,1.01)
*P* for trend			**0.002**	**0.032**
BMI (kg/m^2^)				
< 18.5	269 (3.1%)	33 (4.5%)	1.41 (0.82,2.44)	1.4790.85,2.54)
18.5–23.9	4381 (50.3%)	408 (56.1%)	ref.	ref.
≥ 24	4060 (46.6%)	286 (39.3%)	0.82 (0.65,1.04)	**0.82 (0.67,1.03)**
P for trend			**0.040**	**0.027**
Smoking cigarettes per day				
No	4524 (51.9%)	328 (45.1%)	ref.	ref.
ever smoker	687 (7.9%)	67 (9.2%)	1.20 (0.80,1.80)	1.23 (0.82,1.86)
current smoker	3499 (40.2%)	332 (45.7%)	1.10 (0.85,1.41)	1.11 (0.86,1.43)
≤ 3 pack-years	1529 (17.6%)	134 (18.4%)	1.17 (0.86,1.60)	1.18 (0.86,1.63)
4–6 pack-years	1531 (17.6%)	135 (18.6%)	1.00 (0.73,1.38)	1.02 (0.74,1.40)
> 6 pack-years	1126 (12.9%)	130 (17.9%)	1.19 (0.86,1.66)	1.22 (0.87,1.70)
P for trend			0.319	0.266
Drinking per year				
No	6733 (77.3%)	577 (79.4%)	ref.	ref.
Ever drinker	1438 (16.5%)	118 (16.2%)	1.29 (0.95,1.75)	1.32 (0.97,1.79)
Current drinker	539 (6.2%)	32 (4.4%)	0.80 (0.47,1.35)	0.81 (0.48,1.36)
≤ 180 times	655 (7.5%)	43 (5.9%)	1.09 (0.70,1.68)	1.10 (0.71,1.71)
181–440 times	656 (7.5%)	46 (6.3%)	1.14 (0.73,1.80)	1.12 (0.71,1.77)
> 440 times	666 (7.7%)	61 (8.4%)	1.20 (0.79,1.82)	1.26 (0.83,1.91)
P for trend			0.216	0.172
Only red wine	742 (8.5%)	52 (7.2%)	1.23 (0.81,1.88)	1.21 (0.79,1.86)
Only white liquor	411 (4.7%)	34 (4.7%)	0.88 (0.50,1.53)	0.89 (0.51,1.55)
Only beer	14 (0.2%)	1 (0.1%)	0.54 (0.03,8.52)	0.67 (0.04,10.20)
Red wine+ white liquor	741 (8.5%)	55 (7.6%)	1.13 (0.75,1.69)	1.17 (0.78,1.76)
White liquor + beer	28 (0.3%)	2 (0.3%)	1.35 (0.17,11.10)	1.59 (0.19,13.25)
Red wine+beer	18 (0.2%)	3 (0.4%)	5.02 (0.93,27.09)	**5.47 (1.03,29.18)**
Red wine+beer+ white liquor	23 (0.3%)	3 (0.4%)	2.58 (0.62,10.77)	2.83 (0.67,11.89)

^a^ Adjusted for maternal factors including maternal age, educational attainment, employment status, pre-pregnancy BMI, parity, hypertensive disorders during pregnancy, maternal smoking and family’s average monthly income

^b^ Adjusted for above maternal factors and paternal factors other than the investigated variable

The overall prevalence of PTB was 9.9%. Table 3 presents the associations between paternal factors and PTB. The OR for the highest paternal education was 0.6 (95% CI: 0.49, 0.73) for an association with PTB. Paternal higher education (OR = 0.55, 95%CI: 0.43, 0.71) and higher weight (*P* for trend < 0.0001, were associated with a lower rate of PTB. In addition, father who smoked more than 6 pack-years was associated with PTB (OR = 1.31, 95%CI: 1.07, 1.61). There were no significant associations regarding age, height, or drinking.

**Table 3:** Association between paternal factors and Preterm births by multiple logistic regression analysis

Paternal factors	Term births (***N*** = 9122) (***n***/%)	Pre-term Briths (***N*** = 999) (***n***/%)	OR (95%CI)^**a**^	OR (95%CI)^**b**^
Age				
< 25	691 (7.6%)	118 (11.8%)	ref.	ref.
25 to < 30	3215 (35.2%)	321 (32.1%)	0.97 (0.75,1.25)	1.04 (0.80,1.35)
30 to < 35	3491 (38.3%)	330 (33.0%)	0.85 (0.64,1.13)	0.93 (0.70,1.23)
≥ 35	1725 (18.9%)	230 (23.0%)	0.77 (0.55,1.07)	0.83 (0.60,1.16)
*p* for trend			0.166	0.355
Ethnicity				
Han	8410 (92.2%)	881 (88.2%)	ref.	ref.
Others	712 (7.8%)	118 (11.8%)	1.08 (0.76,1.53)	1.06 (0.74,1.50)
Education years (%)				
Less than high school graduation	1620 (17.8%)	384 (38.4%)	ref.	ref.
high school and community college	3629 (39.8%)	361 (36.1%)	**0.58 (0.48,0.71)**	**0.60 (0.49,0.73)**
high than college	3873 (42.5%)	254 (25.4%)	**0.53 (0.42,0.69)**	**0.55 (0.43,0.71)**
Height (m)				
< 1.71	2767 (30.3%)	354 (35.4%)	ref.	ref.
1.71–1.74	2291 (25.1%)	261 (26.1%)	1.00 (0.84,1.20)	1.02 (0.85,1.22)
≥ 1.75	4064 (44.6%)	384 (38.4%)	0.93 (0.79,1.09)	0.95 (0.81,1.11)
p for trend			0.094	0.169
Weight (Kg)				
< 67.0	2820 (30.9%)	393 (39.3%)	ref.	ref.
67–74.9	2534 (27.8%)	265 (26.5%)	**0.82 (0.69,0.97)**	**0.81 (0.67,0.98)**
≥ 75	3768 (41.3%)	341 (34.1%)	**0.79 (0.68,0.93)**	**0.74 (0.57,0.96)**
p for trend			**0.002**	**0.005**
BMI (kg/m^2^)				
< 18.5	280 (3.1%)	37 (3.7%)	1.03 (0.71,1.48)	1.00 (0.69,1.45)
18.5–23.9	4541 (49.8%)	543 (54.4%)	ref.	ref.
≥ 24	4301 (47.2%)	419 (41.9%)	0.91 (0.79,1.05)	0.92 (0.80,1.06)
*p* for trend			**0.013**	**0.026**
Smoking cigarettes per day				
No	4764 (52.2%)	467 (46.8%)	ref.	ref.
ever smoker	716 (7.9%)	86 (8.6%)	1.07 (0.83,1.39)	1.05 (0.81,1.350
current smoker	3642 (39.3%)	446 (44.6%)	1.11 (0.95,1.30)	1.10 (0.93,1.28)
≤ 3 packyears	1605 (17.6%)	172 (17.2%)	0.99 (0.81,1.20)	0.96 (0.79,1.17)
4–6 packyears	1584 (17.4%)	185 (18.5%)	1.08 (0.89,1.30)	1.06 (0.87,1.28)
> 6 packyears	1169 (12.8%)	175 (17.5%)	**1.32 (1.08,1.62)**	**1.31 (1.07,1.61)**
*p* for trend			0.051	0.065
Drinking per year				
No	7032 (77.1%)	796 (79.7%)	ref.	ref.
ever drinker	1515 (16.6%)	154 (4.9%)	0.91 (0.75,1.10)	0.92 (0.76,1.12)
current drinker	575 (6.3%)	49 (4.9%)	0.80 (0.58,1.09)	0.80 (0.59,1.10)
≤ 180 times	687 (7.5%)	67 (6.7%)	0.90 (0.69,1.18)	0.91 (0.70,1.20)
181–440 times	701 (7.7%)	56 (5.6%)	**0.71 (0.53,0.96)**	**0.71 (0.53,0.96)**
> 440 times	702 (7.7%)	80 (8.0%)	1.03 (0.80,1.32)	1.05 (0.81,1.35)
p for trend			0.828	0.878
only red wine	790 (8.7%)	66 (6.6%)	0.77 (0.59,1.01)	0.79 (0.60,1.03)
only white liquor	438 (4.8%)	44 (4.4%)	0.90 (0.65,1.26)	0.93 (0.66,1.29)
only beer	13 (0.1%)	2 (0.2%)	1.02 (0.21,5.03)	1.04 (0.21,5.19)
red wine+ white liquor	775 (8.5%)	82 (8.2%)	0.95 (0.74,1.22)	0.96 (0.75,1.23)
white liquor + beer	30 (0.2%)	2 (0.2%)	0.93 (0.27,3.18)	0.93 (0.27,3.24)
red wine+beer	20 (0.2%)	2 (0.20%)	0.89 (0.20,3.98)	0.93 (0.21,4.18)
red wine+beer+ white liquor	24 (0.3%)	4 (0.4%)	1.22 (0.41,3.65)	0.93 (0.27,1.23)

^a^ Adjusted for maternal factors including maternal age, educational attainment, employment status, pre-pregnancy BMI, parity, hypertensive disorders during pregnancy, maternal smoking and family’s average monthly income,

^b^ Adjusted for above maternal factors and paternal factors other than the investigated variable

The overall prevalence of SGA was 7.8%. The associations between paternal factors and SGA are shown in Table 4. Paternal education was associated with SGA (OR = 0.61, 95% CI: 0.50, 0.82). Men with a height > 1.75 m had a lower risk of SGA (OR = 0.77, 95% CI: 0.64, 0.92) compared with men with a height < 1.71 m. Compared with infants born with normal weight, the fathers whose BMI was > 24 kg/m^2^ were associated with a lower rate of SGA (OR = 0.60, 95%CI: 0.41,0.86). There were no significant associations regarding age, weight, smoking, or drinking.

**Table 4:** Association between paternal factors and SGA by multiple logistic regression analysis

Paternal factors	AGA (***N*** = 7922) (%)	SGA (***N*** = 785) (%)	OR (95%CI)^**a**^	OR (95%CI)^**b**^
Age				
< 25	628 (7.9%)	101 (12.9%)	ref.	ref.
25 to < 30	2799 (35.3%)	264 (33.6%)	**0.75 (0.56,0.99)**	0.79 (0.60,1.06)
30 to < 35	3009 (38.0%)	257 (32.7%)	**0.69 (0.51,0.94)**	0.75 (0.55,1.02)
≥ 35	1486 (18.8%)	163 (20.8%)	0.74 (0.52,1.06)	0.80 (0.56,1.15)
*p* for trend			0.061	0.133
Ethnicity				
Han	7284 (92.0%)	704 (89.7%)	ref.	ref.
Others	638 (8.1%)	81 (10.3%)	1.00 (0.68,1.48)	1.00 (0.67,1.49)
Education years (%)				
Less than high school graduation	1505 (19.0%)	266 (33.9%)	ref.	ref.
high school and community college	3142 (39.7%)	289 (36.8%)	**0.65 (0.52,0.81)**	**0.68 (0.54,0.86)**
higher than college	3275 (41.3%)	230 (29.3%)	**0.58 (0.43,0.77)**	**0.61 (0.50,0.82)**
Height (m)				
< 1.71	2448 (30.9%)	299 (38.1%)	ref.	ref.
1.71–1.74	2007 (25.3%)	199 (25.4%)	0.87 (0.72,1.05)	0.87 (0.71,1.06)
≥ 1.75	3467 (43.8%)	287 (36.6%)	**0.77 (0.64,0.91)**	**0.77 (0.64,0.92)**
*p* for trend			**0.009**	**0.017**
Weight (kg)				
< 67.0	2545 (32.1%)	322 (41.0%)	ref.	ref.
67–74.9	2205 (27.8%)	213 (27.1%)	0.82 (0.68,0.996)	0.88 (0.72,109)
≥ 75	3172 (40.0%)	250 (31.9%)	0.73 (0.61,0.87)	0.80 (0.60,1.06)
*p* for trend			**< 0.001**	**0.014**
BMI (kg/m^2^)				
< 18.5	247 (3.1%)	40 (5.1%)	1.38 (0.96,1.97)	1.38 (0.96,1.97)
18.5–23.9	4015 (50.7%)	444 (56.6%)	ref.	ref.
≥ 24	3660 (46.2%)	301 (38.3%)	0.82 (0.70,0.96)	0.83 (0.70,0.97)
*p* for trend			**0.001**	**0.001**
Smoking cigarettes per day				
No	4097 (51.7%)	378 (48.2%)	ref.	ref.
ever smoker	630 (8.0%)	63 (8.0%)	0.95 (0.71,1.27)	0.95 (0.71,1.27)
current smoker	3195 (40.3%)	344 (43.8%)	0.99 (0.83,1.17)	0.98 (0.82,1.17)
≤ 3 pack-years	1408 (17.8%)	137 (17.5%)	0.91 (0.74,1.13)	0.91 (0.73,1.13)
4–6 pack-years	1382 (17.5%)	147 (18.7%)	0.99 (0.80,1.23)	0.98 (0.79,1.22)
> 6 pack-years	1035 (13.1%)	123 (15.7%)	1.07 (0.85,1.35)	1.06 (0.84,1.35)
*p* for trend			0.702	0.737
Drinking per year				
No	6128 (77.4%)	610 (77.7%)	ref.	ref.
ever drinker	1300 (16.4%)	142 (18.1%)	1.10 (0.90,1.34)	1.11 (0.91,1.36)
current drinker	494 (6.2%)	33 (4.2%)	0.69 (0.47,0.995)	0.69 (0.47,0.992)
≤ 180 times	583 (7.4%)	57 (7.3%)	1.00 (0.75,1.34)	1.02 (0.76,1.37)
181–440 times	585 (7.38%)	63 (8.03%)	1.08 (0.82,1.43)	1.08 (0.81,1.43)
> 440 times	626 (7.9%)	55 (7.0%)	0.88 (0.65,1.12)	0.88 (0.66,1.19)
*p* for trend			0.456	0.464
Only red wine	670 (8.5%)	610 (77.7%)	0.97 (0.74,1.29)	0.99 (0.75,1.31)
Only white liquor	380 (4.8%)	64 (8.2%)	0.83 (0.57,1.21)	0.83 (0.56,1.21)
Only beer	14 (0.2%)	32 (4.1%)	0.54 (0.07,4.28)	0.60 (0.08,4.87)
Red wine+ white liquor	663 (8.4%)	1 (0.1%)	1.10 (0.85,1.44)	1.11 (0.85,1.44)
White liquor + beer	26 (0.3%)	71 (9.0%)	0.71 (0.16,3.12)	0.74 (0.17,3.25)
Red wine+beer	18 (0.2%)	2 (0.3%)	1.01 (0.22,4.57)	0.93 (0.20,4.31)
Red wine+beer+ white liquor	23 (0.3%)	3 (0.4%)	1.16 (0.34,3.95)	1.14 (0.33,3.97)

^a^ Adjusted for maternal factors including maternal age, educational attainment, employment status, pre-pregnancy BMI, parity, hypertensive disorders during pregnancy, maternal smoking and family’s average monthly income

^b^ Adjusted for above maternal factors and paternal factors other than the investigated variable

## Discussion

To our knowledge, the present study is the first to comprehensively examine the effect of paternal factors on the risk of a wide range of adverse perinatal outcomes (LBW, PTB, and SGA) in a Chinese population. It supports the hypothesis that paternal lower education and lower weight are associated with a higher incidence of PTB and SGA. Moreover, high paternal BMI (> 24 kg/m^2^) is associated with a low risk of LBW, PTB, and SGA, our study also suggested that paternal smoking is associated with a high risk of PTB. The previous studies that investigated the association between paternal age and adverse birth outcome provided conflicting results. It was reported significant associations between younger paternal age and PTB [3], whereas others [4] found that advanced paternal age (> 45 or > 50 years) increased the risk of PTB. In the present study, no association was found between age and PTB, LBW, and SGA. A comparison of those studies is a challenge because of the different age cut-offs that were used, as well as the different reference age groups. Although most previous studies [4, 5] had a large sample size (> 70,000) based on national or state databases, some key covariates could not be assessed (such as obstetric complication and maternal smoking status), and significant biases may affect the selection of controls.

Habib et al. [22] indicated that paternal SES characteristics appear to have a stronger influence on perinatal mortality than maternal SES characteristics, which may reflect social and cultural conditions that need to be considered by policymakers in developing countries. Compared with men who had a high school education (≥16 years), higher education was associated with lower odds of PTB and SGA in the present study. The possible reasons are not clear. Nevertheless, those results are globally supported by previous studies [8–10].

A recent study documented that maternal height contributes to the intrauterine environment and to infant growth and gestational age, which may overcome the influence of paternal genes. In order to rule out the effect of maternal height, we stratified the subjects by maternal height but did not found any interaction between paternal height and adverse birth outcomes (*P* > 0.05), but fathers who were taller were associated with a decreased risk of LBW (*P* for trend = 0.0013), independently from maternal height. This is supported by the literature [12], but there are conflicting results [6]. The effect is presumed to be heritable through the paternal germline [23].

Regarding paternal weight and BMI on birth weight, we observed an association between paternal weight and LBW, as supported by previous studies [6, 23]. Unlike the study by McCowan et al. [24], we found that paternal underweight may increase the risk of SGA, independent of maternal BMI. Possible reasons for this discrepancy is the use of different birth weight standards and paternal BMI cut-off points. Indeed, the BMI categories in China are different from those of other parts of the world. Interesting, we also found that paternal weight is an independent factor of PTB, even after adjustment for maternal weight.

As for the paternal lifestyle habits, although previous studies [13] paid more attention to paternal smoking and offspring’s birth weight, the present study was inconsistent with studies by Ko et al. [14], Inoue et al. [15], and Fan et al. [16], which reported that paternal smoking did not have any significant relationship with adverse birth outcomes. The present study found that heavy paternal smoking was associated with a significant increased risk of PTB, but we could observe a slim dose-response relationship, nor associations with LBW and SGA. Paternal smoking was reported to be associated with pregnancy complications [14], but the exact mechanisms are unknown. Paternal smoking may affect birth outcomes through two possible mechanisms: a genetic effect on sperm and maternal passive smoking. Indeed, smokers have an increased frequency of abnormal sperm morphology, decreased sperm penetration, and significantly higher mutagen levels in urine [25, 26]. In the present study, paternal smoking was correlated (r = 0.32) with maternal passive smoking. Therefore, it is impossible to separate these two mechanisms based on the available data.

In addition, our study found no significant association between paternal alcohol consumption and adverse birth outcomes, which was similar to the literature [16]. Up to now, only one study [17] found a positive association with alcohol, but it should be noted that the population of this study was identified by selecting spouses of mothers who were regular or occasional alcohol consumers before and during pregnancy (half in each group), enriching the sample for male alcohol consumers as a result of spousal concordance. In contrast, few women consumed alcohol before deliveries or during pregnancy (0.2%) in the present study.

The etiologies of adverse birth outcomes are multifactorial and not completely understood yet [27–35]. In the present study, we collected the paternal characteristics that could be potential risk factors for adverse birth outcomes and included them in the final model combined with maternal characteristics. No clear mechanism by which paternal factors could lead to an increased risk for adverse birth outcomes have yet been postulated. Some biological evidence showed that the placenta could express some genes from paternal origins [36], and these potentially harmful mutations in genes of placentation may be more frequent among immature men [37]. In view of the strong association between paternal and maternal age in the present study (younger fathers were more likely to have younger partners), both of them maybe have not reached their full biological maturity or have an unplanned birth. On the other hand, compared with older fathers, young fathers frequently have a low level of education and have difficulty coping with the knowledge of pregnancy [38]. Moreover, young fathers usually have poor financial support and employment status and would show signs of clinical depression and stress [39]. Last but not least, different environments, nutrition, and lifestyle risk factors may explain the association of adverse birth outcomes. For now, we cannot distinguish whether the paternal factors directly or indirectly influencing the birth outcomes through interactions with maternal factors, those originating from the paternal genetic effect, and those from the environment.

The interpretation of the results of this study must take into account its limitations. First, in relation to the ascertainment of exposure, the data regarding the father were mostly self-reported except for height and weight, therefore prone to recall bias. Second, selection bias might have occurred as some fathers were less willing to co-operate in the birth cohort study or were absent when we interviewed the women. A meta-analysis of studies that used biochemical measurements to validate self-reported smoking behavior found that the self-reporting of smoking was generally reliable [40]. Unfortunately, we could not collect the sociodemographic characteristics of those who refused to participate. Additionally, the exclusion of twin pregnancy and stillbirth might account for part of selection bias. Third, our study was a hospital-based study, which might impact generalizability, but the rates of LBW (7.2%), PTB (9.2%), and SGA (7.8%) in our study population were within the reported ranges for LBW (5.1–9.0%) [41], PTB (2.3–10.3%) [4], and SGA (7%) [42].

Despite these shortcomings, our study has some strengths. The first is the exclusion of multiple births and stillbirths, minimizing the potential differences in health effects of birth weight and gestation age. Secondly, all participants were Chinese, minimizing differences in genetic susceptibility to birth weight and gestation age. Third, we collected and controlled potential confounding factors in the analysis. Information on birth outcomes and maternal complications during pregnancy was obtained from the medical records, minimizing misclassification.

## Conclusions

This is the first study in China to show low paternal education and low weight are associated with a higher incidence of PTB and SGA. Paternal overweight was associated with lower LBW, PTB, and SGA. Paternal smoking is associated with high risk of PTB. These associations could provide clues to the etiology of these conditions. Further scientific and epidemiologic studies are needed to elucidate the mechanism of how paternal factors influence fetal developmental processes.

## Supplementary Information

**Additional file 1: Supplementary File 1**. Questionnaire

## Data Availability

The datasets used and/or analysed during the current study are available from the corresponding author on reasonable request.

## Abbreviations

BMI: Body mass index
CI: Confidence interval
LBW: Low birth weight
OR: Odds ratio
PTB: Preterm birth
SGA: Small for gestational age
