# Supplementary material for: Paternal factors and adverse birth outcomes in Lanzhou, China
Source: BMC Pregnancy Childbirth. 2021 Jan 6;21:19. doi: 10.1186/s12884-020-03492-9 (PMC7789361; doi:10.1186/s12884-020-03492-9)
Supplement: Supplementary file 1 — Additional file 1: Supplementary File 1. Questionnaire [file 12884_2020_3492_MOESM1_ESM.pdf]

China Birth Cohort Study – Pregnant Women's Health Questionnaire

2010-2012

Gansu Provincial Maternity and Child Care Hospital

Yale University School of Public Health

A volume ----Health information of pregnant women ----Personal Information

Respondent's ID____Inpatient department _________Bed number
Hospital admission number____ Doctor at admission ____
Doctor in charge ____ Nurse at admission ____
Investigator ____

001. Respondent's name_________

002. Your identification card number _________

003. Your birthday (solar/lunar calendar) (Solar calendar is the priority if remembered) ___

004. Your nation ______

005. Your living address

Province__________ City ________

District_________ Road _________

Door number_______ Home phone number_________

Mobile phone number__________

006. Current work unit (If retired, record the past work unit)____

007. Work address

Province____ City____
District____ Road ____
Door number____ Business phone number____
E-mail____

008. Name of child's father ________

009.His identification card number____

010. His birthday (solar/lunar calendar) (solar calendar is the priority if remembered)
Year: ___Month: ______ Day: ______

011. His nation____

012. His current work unit (If retired, record the past work unit)
His work content in details _____

013. His work address
Province_________ City_________District____Road____
Door number____ His business phone number____
His mobile phone number____
014. In order to conveniently follow up you and your children's health status , please choose one of your relatives (father, mother, brothers, or sisters, etc.) as a contact person and record his/her name, home address, home phone number, work unit and business phone number.

____

015. Please provide the name, home address, home phone number, work unit and business phone number of another relative or friend
____

016． Living address with longest time during pregnancy
City ____ District ____ Road name____

Pregnant women's health investigation
Part I. Basic Information

A1. Date year:____ month:__day:______

A2. At what age did you first live in this city? (if you were born here, record 0)

_____

A3. What is your education level?

1——Without formal education)	*2—-Elementary school*
3——Middle school	*4—High school/technical school*
5——Community college)	*6——College*
7——Graduate school)	*9—— Unknown*

A4. Last year (2009), how much is your family's average monthly income (including all sources) per person?

1——< 1000 Yuan *2——1000-2000 Yuan*
3——2000-3000 Yuan *4——3000-4000 Yuan*
5——4000-5000 Yuan *6——>5000 Yuan*
7——Unknown

A5. Did you have prepregnancy health education?
0——No *1——Yes*

A6. Your gestational week when you took the first perinatal examination __________

A7. Total times of perinatal examinations ______

Part II. Reproductive History

B1. Age of menarche ___________

B2. In one year before pregnancy, was your menstruation regular?
0——Regular (skip to B3) *1——Not regular*

If it was treated with medication, what was the name?

B3. First date of your last menstrual period before pregnancy year:____ month:____day:____

B4. Expected date of childbirth year:_____ month:______day:____

B5. Has vaginal haemorrhage been seen during pregnancy? (bloody show before delivery is not included)
0——No
1——Yes, slightly
From which week to which week since pregnancy

2——Yes, severe
From which week to which week since pregnancy

If threatened abortion, please fill out the table below.

Medicine name	*Frequency of intake*	*Gestational week when medicine was taken for the first time*	*How many days did you actually take the medicine*

B6. Did you have nausea since pregnancy?

0——No

1——Yes, slightly

From which week to which week since pregnancy

2——Yes, severe

From which week to which week since pregnancy

B7. Have you had amniocentesis test for the pregnancy of index?

0—— No 1——Yes

Reason:

Gestational week when the test was taken

Diagnosis results

B8. Since pregnancy, had doctor ever told you that you had gestational hypertension ?

0——No (skip to B11) *1——Yes*

B9. Diagnosis time of gestational hypertention

year:____ month:___ day:

B10.Have you ever received therapies due to gestational hypertention? (multiple choices)

0—— No *1——Yes, dietary restriction (i.e. non-salt dietary)*

2——Yes, stay in bed *3——Yes, hospitalized care*

4——Yes, drug therapy *5——Yes, others, please specify*

B11. Since this pregnancy, did doctor ever tell you that you had preeclampsia?

0——No (skip to B14) *1——Yes*

B12. Diagnosis time of preeclampsia

B13. Have you ever received therapies due to preeclampsia?(multiple choices)

0——No *1——Yes, dietary restriction (i.e. non-salt dietary)*
2——Yes, stay in bed *3——Yes, hospitalized care*
4——Yes, drug therapy *5——Yes, others, please specify*

B14. Since this pregnancy, did doctor ever tell you that you had eclampsia?

0——No (skip to B14) *1——Yes*

B15 Diagnosis time of eclampsia

B16.Have you ever received therapies due to eclampsia?(multiple choices)

0——No *1——Yes, hospitalized care*
2——Yes, drug therapy *3——Yes, others, Please specify*

B17-B23.elow are the questions about contraceptive methods. Please indicate us which method you have used before and the way of using it.

contraceptive methods	*None use*	*using*					
		Name of the contraceptive medicine/device	*Date of first time use*	*Date of last time use*	*How many months were actually used*	*How many months were used in total in one year before pregnancy*	*Whether it was the last kind of contraceptive method used before pregnancy*
B17。Intrauterine device (IUD)							
B18. Long acting oral contraceptive medication							
B19.hort							

acting oral contraceptive medication							
B20. Emergency Contraception Pills (ECP)							
B21. Norplant implants							
B22. Condoms used by spouses							
B23. Others							

B24. Have you ever been diagnosed with or treated for infertility?

0——No (skip to B31) *1——Yes*

B25. What kind of infertility were you diagnosed with? (i.e. tubal obstruction, anovulatory infertility, etc.)

B26. Age when diagnosed with infertility

B27. Have you ever received surgeries to treat your infertility? (exclude IVF)

0—— No (skip to B31) *1——Yes*

B28. Name of the surgery?

B29.In one year before this pregnancy, did you ever take medicines to treat infertility?

0—— No (skip to B31) *1——Yes*

*B30. Name of the medicine to treat infertility(ie. steroid hormone,etc)*____________

B31.Was the child's father ever diagnosed with or treated for infertility? 0

0——No (skip to B38) *1——Yes*

B32. What kind of infertility was he diagnosed with? (i.e. oligoasthenozoospermia, bateriospermia, etc.)

____

B33. Diagnosis time for infertility____________

B34.Did he ever receive surgeries in order to treat his infertility? (exclude transepithelial sperm aspiration)
0——No (skip to B38) *1——Yes*

B35. Name of the surgery?____________________

B36. In one year before pregnancy, did your child's father ever take medicine in order to treat the infertility?
0—— No (skip to B38) *1——Yes*

B37. Name of the medicine?____________

B38-B41.Did you ever receive hormone therapies and how long was the total treatment time?

diseases	*Untreated*	*treated*					
		Name of the medicine	*Time of first medicine intake*	*Time of last medicine intake*	*Duration of use*	*How many months did you intake medicines in one year before your pregnancy?*	*How many months did you intake medicines during your pregnancy? (If none, record 0.)*
B38. irregular menstruation							
B39. Endometriosis							
B40. whelk							
B41. Other diseases							

B42. Is your pregnancy a natural pregnancy?

0——No *1——Yes (skip to B47)*

B43.If it was assisted conception, what was the way of the conception?

1— Artificial Insemination *2—First generation of In Vitro Fertilization (IVF-ET)*

3—Second generation of In Vitro Fertilization (ICSI) *4— Third generation of In Vitro Fertilization (PGD)* *5——Frozen embryo transfer conception*

B44.How many periods of the In Vitro Fertilization therapies have you received?____________

B45. How many times had you got pregnant?

Pregnancy___Live birth __

Still birth (gestational weeks ≥28) 1 times

If yes, reason of stillbirth Hypertensive disorder complicating pregnancy

Spontaneous abortion __times Induced abortion____times Ectopic pregnancy____times__

B46. In the first three months of the pregnancy, did you ever have fever?

0——No *1——Yes*

If yes, the highest temperature was

Duration days

Medicine	*Dosage and Frequency*	*Gestational week of first time medicine intake*	*Total days of medicine intake*

Part III. Medical history

C1-C22 Have you ever been diagnosed these diseases below before pregnancy

	Disease name	*Have you ever been diagnosed?*		*Have you ever been treated for this disease in the one year before your pregnancy?*		*Have you ever been treated for this disease during your pregnancy?*	
Cardiovascular	*C1 Hypertension*	*0-No*	*1-Yes*	*0-No*	*1-Yes*	*0-No*	*1-Yes*

diseases	*C2 Coronary heart disease*	*0-No*	*1-Yes*	*0-No*	*1-Yes*	*0-No*	*1-Yes*
	C3 Congenital Heart disease	*0-No*	*1-Yes*	*0-No*	*1-Yes*	*0-No*	*1-Yes*
	C4. Other cardiovascular diseases	*0-No*	*1-Yes*	*0-No*	*1-Yes*	*0-No*	*1-Yes*
respiratory diseases	*C5. Bronchial asthma*	*0-No*	*1-Yes*	*0-No*	*1-Yes*	*0-No*	*1-Yes*
	C6. Chronic bronchitis	*0-No*	*1-Yes*	*0-No*	*1-Yes*	*0-No*	*1-Yes*
	C7. Other respiratory diseases	*0-No*	*1-Yes*	*0-No*	*1-Yes*	*0-No*	*1-Yes*
renal diseases	*C8. Urinary tract calculi*	*0-No*	*1-Yes*	*0-No*	*1-Yes*	*0-No*	*1-Yes*
	C9. Urinary system infection	*0-No*	*1-Yes*	*0-No*	*1-Yes*	*0-No*	*1-Yes*
	C10. Other Urinary system diseases	*0-No*	*1-Yes*	*0-No*	*1-Yes*	*0-No*	*1-Yes*
endocrine diseases	*C11. Diabetes*	*0-No*	*1-Yes*	*0-No*	*1-Yes*	*0-No*	*1-Yes*
	C12. Hyperthyroidism	*0-No*	*1-Yes*	*0-No*	*1-Yes*	*0-No*	*1-Yes*
	C13. Hypothyroidism	*0-No*	*1-Yes*	*0-No*	*1-Yes*	*0-No*	*1-Yes*

	C14. Other endocrine diseases	*0-No*	*1-Yes*	*0-No*	*1-Yes*	*0-No*	*1-Yes*
blood diseases	*C15. Iron-deficiency anemia*	*0-No*	*1-Yes*	*0-No*	*1-Yes*	*0-No*	*1-Yes*
	C16. Other blood diseases	*0-No*	*1-Yes*	*0-No*	*1-Yes*	*0-No*	*1-Yes*
virus hepatitis	*C17. Viral hepatitis B*	*0-No*	*1-Yes*	*0-No*	*1-Yes*	*0-No*	*1-Yes*
	C18. Viral hepatitis C	*0-No*	*1-Yes*	*0-No*	*1-Yes*	*0-No*	*1-Yes*
	C19. Other virus hepatitis	*0-No*	*1-Yes*	*0-No*	*1-Yes*	*0-No*	*1-Yes*
psychological diseases	*C20. Depression*	*0-No*	*1-Yes*	*0-No*	*1-Yes*	*0-No*	*1-Yes*
	C21. Schizophrenia	*0-No*	*1-Yes*	*0-No*	*1-Yes*	*0-No*	*1-Yes*
	C22. Other psychological diseases	*0-No*	*1-Yes*	*0-No*	*1-Yes*	*0-No*	*1-Yes*

C23-C36 During pregnancy, had you ever used medication to treat the following symptoms or diseases

symptoms	*0-No 1-Yes*	*Medicine name*	*Gestational week of*	*Total weeks of*

			first use	*using medicines*
C23. Nausea, vomiting				
C24.Edema				
C25. Malnutrition				
C26. Somnolence				
C27. Tension, anxiety				
C28. Depression				
C29. Headache or other pains				
C28. Depression				
C29. Headache or other pains				
C30. Infection				
C31. Acne or other skin diseases				
C32. Leg cramp				
C33. Astriction				
C34. Indigestion or heartburn				
C35. Stomachache,				

belching or ulcer				
C36. Hypertension				

Part XI. Medical Records

H1.Diagnosis at discharge __________________________

H2. Auxiliary examination results

Blood routine: _____ *N%：* *Granulocyte count：* _____ *X10⁹/L；* *Total lymphocyte count：* _____*X10⁹/L*

*RBC*_____ *X10⁹/L* *Hb* _____ *g/L* *HCT :* _____ *%* *PLT* _____ *X10⁹/L*

Urine routine*:PRO (qualitative)* _____ *Quantitative*_____ *g/24h;* *urine GLU*_____ *UBG* _____

*Urobilirubin*_____

Coagulation:

*PT*_____ *TT* _____ *APTT*_____ *FIB* _____ *D-dimer*_____

Liver function

ALT _____ *AST* _____ *ALP*_____ *r-GT* _____ *TB* _____ *DB* _____ *Cholic acid* _____

Total protein (TP) _____ *albumen* _____

Renal function

BUN _____ *Cr*_____ *Uric acid (UA)* _____ *Blood glucose*_____

Blood lipid

*TC*_____ *TG*_____ *HDL*_____ *LDL* _____

Fetal heart monitoring

Umbilical cord blood flow ratio:

_____

H3.If there is any other abnormal result in other special examinations, please indicate (for example, bone marrow puncture, Chest X-radiography, CT)

H4 Ways of delivery

1——Vaginal delivery *2——Vaginal trial production* *3——Cesarean section*

If a surgery was involved (assisted vaginal delivery, or cesarean section), please indicate the reason

Volume B

Part IV. Life Style Factors

D1. Have you ever smoked (at least one cigarette per day lasting for one month)?

0—— No (skip to D8) *1——Yes*

D2. From what age did you start smoking frequently?____

D3. When you smoked frequently, how many cigarettes per day did you smoke? ____

D4. Do you smoke now?

0——No *1—— Yes (skip to D6)*

D5. At what age did you stop smoking?____

D6. How long did you smoke frequently?____

Year____ *Month____*

D7. In the one year before your pregnancy, how many cigarettes per day did you smoke? ____

D8. In the first 13 weeks of this pregnancy, have you ever smoked or been exposed to a smoking environment?

0— No (skip to D14) *1——Yes*

D9. In the first 13 weeks of this pregnancy, how many days have smoked?

Usually, how many cigarettes per day did you smoke?____

D10. In the first 13 weeks of this pregnancy, did your husband smoke in front of you

0—— No (skip to D12) *1——Yes*

D11. How many hours per day had your husband smoked in front of you? ____ hour

D12. In the first 13 weeks of this pregnancy, was there anyone else who smoked in front of you?_

0—— No (skip to D14) *1——Yes*

D13. In the first 13 weeks of this pregnancy, How many hours per day had someone else smoked in front of you?

_____hours

D14. In the second trimester, have you ever smoked or be exposed to a smoking environment?

0—— No (skip to D20) *1——Yes*

D15. How many days have you ever smoked during the second trimester?

How many cigarettes per day did you smoke?____

D16. Did your husband smoke in front of you during the second trimester?

0—— No (skip to D18) *1——Yes*

D17. How many hours per day had your husband smoked in front of you? ______hours

D18. During the second trimester, was there anyone else who smoked in front of you? ______

D19. How many hours per day soemone else smoked in fornt of you during the second trimester?

______hours

D20. During the third trimester, have you ever smoked or be exposed to a smoking environment?

0—— No (skip to D26) *1——Yes*

D21. How many days did you smoke during the third trimester?

How many cigarettes per day did you smoke?

D22. Did your husband smoke in front of you during the third trimester?

0——No (skip to D24) *1——Yes*

D23. How many hours did your husband smoke in front of you?

hours

D24. During the third trimester, was there anyone else who smoked in front of you?

0——No (skip to D26) *1——Yes*

D25. How many hours per day someone else had smoked in front of you during the third trimester?

______hours

D26. Before this pregnancy, did you often drink alcohol?

0—— No (skip to D32) *1—Yes*

D27. From what age did you begin to drink alcohol?____

D28. For each type of alcohol, how many times per week did you drink? How many liangs (50g) every time?

type of alcohol	times per week	Liang per time	type of alcohol	times per week	Liang per time
Beer			Red wine		
Champagne			White wine		
Yellow wine			Liquor with low alcohol (below 40°)		
Rice wine			Liquor with high		

			alcohol (over 40°)		

D29. Do you still often drink alcohol now?
0——No *1—— Yes(skip to 31)*

D30. At what age did you stop frequently drinking alcohol? ____

D31. How long have you been drinking alcohol?
Year____ *Month____*

D32. During the first trimester, did you often drink alcohol? (at least one time per week in average)
0——No (skip to D34) *1——Yes*

D33. For each type of alcohol, times per week and liangs (50g) per time did you drink during the first trimester?

type of alcohol	*times per week*	*Liang per time*	*type of alcohol*	*times per week*	*Liang per time*
Beer			*Red wine*		
Champagne			*White wine*		
Yellow wine			*Liquor with low alcohol (below 40°)*		
Rice wine			*Liquor with high alcohol (over 40°)*		

D34. During the second trimester, did you often drink alcohol? (at least one time per week in average)
0——No (skip to D36) *1——Yes*

D35. For each type of alcohol, times per week and liangs (50g) per time did you drink during the 2nd trimester?

type of alcohol	*times per week*	*Liang per time*	*type of alcohol*	*times per week*	*Liang per time*
Beer			*Red wine*		
Champagne			*White wine*		

Yellow wine			*Liquor with low alcohol (below 40°)*		
Rice wine			*Liquor with high alcohol (over 40°)*		

D36. During the third trimester, did you often drink alcohol? (at least one time per week in average)

0——No (skip to D38) *1——Yes*

D37. For each type of alcohol, times per week and liangs (50g) per time did you drink during the third trimester?

type of alcohol	*times per week*	*Liang per time*	*type of alcohol*	*times per week*	*Liang per time*
Beer			*Red wine*		
Champagne			*White wine*		
Yellow wine			*Liquor with low alcohol (below 40°)*		
Rice wine			*Liquor with high alcohol (over 40°)*		

D38. Before this pregnancy, did you often drink tea? (at least three times per week)

0—— No (skip to D42) *1——Yes*

D39. From what age did you start drinking tea frequently? ____

D40.What kind of tea do you mainly drink? (Choose one type)

1——Green tea *2——Black tea*
3——Oolong tea *4——Scented tea*
5——half black and half green tea *6——half scented tea and half green tea*
7——half scented tea and half red tea *8——Others*

D41. How long have you been drinking tea?

Year____ *Month____*

D42 During the first trimester, did you often drink tea? (at least three times per week)

0—— No (skip to D44) *1——Yes*

D43. What kind of tea do you mainly drink? (Choose one type)

1——Green tea *2——Black tea*
3——Oolong tea *4——Scented tea*
5——half black and half green tea *6——half scented tea and half green tea*
7——half scented tea and half red tea *8——Others*

D44. During the second trimester, did you often drink tea? (at least three times per week)

0—— No (skip to D46) *1——Yes*

D45. What kind of tea do you mainly drink? (Choose one type)

1——Green tea *2——Black tea*
3——Oolong tea *4——Scented tea*
5——half black and half green tea *6——half scented tea and half green tea*
7——half scented tea and half red tea *8——Others*

D46. During the third trimester, did you often drink tea? (at least three times per week)

0——No (skip to D48) *1——Yes*

D47. What kind of tea do you mainly drink? (Choose one type)

1——Green tea *2——Black tea*
3——Oolong tea *4——Scented tea*
5——half black and half green tea *6——half scented tea and half green tea*
7——half scented tea and half red tea *8——Others*

D48. In the one year before your pregnancy, did you often take vitamins or mineral supplements?

0——No (skip to D53)
1——Yes

Name	*How many weeks in total did you take vitamins or mineral supplements*	*Times per week of taking vitamins or mineral supplements*
D49		
D50		
D51		
D52		

D53. During the first trimester, did you often take vitamins or mineral supplements?

0——No (skip to D58)

1——Yes

Name	*How many weeks in total did you take vitamins or mineral supplements*	*Times per week of taking vitamins or mineral supplements*
D54		
D55		
D56		
D57		

D58. During the second trimester, did you often take vitamins or mineral supplements?

0——No (skip to D63)
1——Yes

Name	*How many weeks in total did you take vitamins or mineral supplements*	*Times per week of taking vitamins or mineral supplements*
D59		
D60		
D61		
D62		

D63. During the third trimester, did you often take vitamins or mineral supplements?

0——No (skip to E1)
1——Yes

Name	*How many weeks in total did you take vitamins or mineral supplements*	*Times per week of taking vitamins or mineral supplements*
D6		
D65		
D66		
D67		

Part V. Physical Activities

E1. In the one year before your pregnancy, did you attend any physical activities (at least three times per week, and thirty minutes per time)?

0——No (skip to E3) *1——Yes*

E2. How many hours per week during the one year before pregnancy ?   take walk for __hours__physical activities for__hours

E3. During the first trimester, did you attend any physical activities (at least three times per week, and thirty minutes per time)?

0——No (skip to E5)   1——Yes

E4. How many hours per week during the first trimester?

take walk for_____hours__physical activities for____hours

E5. During the second trimester, did you attend any physical activities?

0—— No (skip to E7)   1——Yes

E6. How many hours per week during the second trimester?

take walk for _____hours__physical activities for____hours

E7. During the third trimester, did you attend any physical activities?

0——No (skip to E9)   1——Yes

E8.How many hours per week during the third trimester?

take walk for ____hours__physical activities for___hours

E9. During this pregnancy, how many hours per day do you use computer? ____ hour

E 10. During this pregnancy, how many hours per day do you watch TV? ____ hours

E11. During this pregnancy, when you watched TV or used computer, did you use radiation proof clothes?

0——No   1——Occasionally

2——Often   3——Always

Part VI. Occupation

F1. During this pregnancy, what is the name of your work unit (if you do not have a job, record the last one that you had and the end time.)

Name of the institution where you work none

work address ____________________

How many hours do you work per day____________________________

F2. What is the content of you work? (exposure to chemicals/ radiation, high temperature, or noise)________

F3. When did you start this job?____________

F4. Is this the job which you have been worked for the longest time?

0——No *1——Yes(skip to G1)*

F5. The name of the institution where you have worked for the longest time________

F6. What is the content of you work? (exposure to chemicals/ radiation, high temperature, or noise)____________

F7. When did you start this job? (including the time before the pregnancy)____________

F8. When did you stop this job?____________

Part VII. Information of Husband (father of the child)

G1. What is the education level of the Father?

1——without formal education *2——Primary school*
3——Junior high school *4——Senior high school/technical secondary school*
5——Junior college *6——University*
7——Master and the above *9——Unknown*

G2. The Father's weight _____jin

G3. The Father's height (cm) _____cm

G4. Did the Father smoke (at least one cigarette per day lasting for one month)? _

0—No (skip to G10) *1——Yes*

G5. At what age did the Father smoke frequently? ______years old

G6. When the Father smoked frequently, how many cigarettes per day did the Father smoke?

_____cigarettes

G7. Does the Father often smoke now?

0——No *1—— Yes(skip to G9)*

G8. When did the Father stop smoke frequently?

G9. How long time did the Father smoke frequently?

Years ____

G10. Did the Father often drink alcohol? (at least one time per week in average)

0—— No (skip to H1) *1——Yes*

G11. At what age did the Father start drinking alcohol? _____years old

G12. When the Father drank frequently, usually how many times did the Father drink per month? _______times/month

G13. When the Father drank frequently, what kinds of alcohol did the Father drink mainly?

____

G14. Does the Father often drink now?

0——No *1—— Yes(skip to G16)*

G15. At what age did the Father stop frequently drinking alcohol?____

G16. How long time did the Father drink frequently?

Year____________ *Month____________*

Part VIII. Development history and body measurement

H1. Your weight when you were born (0.5kg) __________jin

H2. Were you raised up by breast feeding?

0—No *1——Yes* *9——Unknown*

H3. Before pregnancy, how many Jin(500g) was your average weight? ____jin

H4. When you have been pregnant for 3 months, how many Jin was your average weight? ___jin

H5. Before delivery, how many Jin was your average weight?___jin

H6. Your height (cm)_____cm

Part IX Residencial History

	（1）Current residence	（2）Previous residence	（3）The one before previous residence
I1. Name of district and street	District	District	District
	Street	Street	Street
I2. Date of starting to live			
I3. What kinds of fuel do you usually use for cooking in this residence?	1——Coal gas or natural gas	1——Coal gas or natural gas	1——Coal gas or natural gas
	2——briquettes	2——briquettes	2——briquettes
	3—Others	3—Others	3—Others
I4. Do you usually cook with high temperture oil at beginning?	0—— No 1——Yes	0—— No 1——Yes	0—— No 1——Yes
I5. Do you use ventilation in the kitchen?	0—— No 1——Yes	0—— No 1——Yes	0—— No 1——Yes

Part X Diet

In the below, the status of diet in the one year before your pregnancy is wished to be known.

J1. How was the food that you often have? ________

In the one year before your pregnancy

1——Very salty *2——Relatively salty*
3——Not very salty *4——Not salty at all*

In the one year before your pregnancy

1——Very spicy *2—- Relatively spicy*
3—— Not very spicy *4——Not spicy at all*

In the one year before your pregnancy _

1—— Very sweet *2—— Relatively sweet*
3—— Not very sweet *4——Not sweet at all*

After pregnancy _

1——Very salty *2——Relatively salty*
3——Not very salty *4——Not salty at all*

After pregnancy _

1——Very spicy *2—— Relatively spicy*
3——Not very spicy *4——Not spicy at all*

After pregnancy _

1——Very sweet *2—— Relatively sweet*
3——Not very sweet *4——Not sweet at all*

J2. The two most common ways of cooking that your family use

In the one year before your pregnancy _

1——Deep fried *2——Braised*
3——Pan fried *4——Steaming*
5——Simmer（Bland） *6——Other, please indicate*

After pregnancy __

1——Deep fried *2——Braised*
3——Pan fried *4——Steaming*
5——Simmer（Bland） *6——Other, please indicate*

J3. Do you often have garlic?

In the one year before your pregnancy

1——Never have *2——Seldom have*
3——Often have *4——Unknown*

After pregnancy _

1——Never have *2——Seldom have*
3——Often have *4——Unknown*

J4. Which kinds of cooking oil did your family eat?

in the one year before your pregnancy _

1——Canola oil *2——Soybean oil*
3——Salad oil *4——Peanut oil*
5——Sesame oil *6——Lard*
7——Others

After pregnancy

1——Canola oil *2——Soybean oil*
3——Salad oil *4——Peanut oil*
5——Sesame oil *6——Lard*
7——Others

Please recall in the one year before your pregnancy and since this pregnancy, respectively, have you ever had the following food (if no, fill in 0), and estimate the average amount and times of having these food

Food name	In the one year before pregnancy					During the first trimester					During the second trimester					During the third trimester				
	Average amount	times				Average amount	times				Average amount	times				Average amount	times			
		None fill in 0	Every day	Every week	Every month		None fill in 0	Every day	Every week	Every month		None fill in 0	Every day	Every week	Every month		None fill in 0	Every day	Every week	Every month
J5. Rice(liang)																				
J6. Wheat flour(liang)																				
J7. Coarse cereals(liang)																				
J8.Pork(liang)																				
J9.Beef(liang)																				
J10.Lamp(liang)																				
J11.Chicken, duck(liang)																				
J12.Freshwater fish(liang)																				
J13.Saltwater fish(liang)																				
J14. Shrimps, crabs, seashells(liang)																				
J15. Fresh milk(ml)																				

J16. Milk powder(g)																				
J17.Yogurt (ml)																				
J18. Eggs																				
J19. Soybean milk(ml)																				
J20 Tofu and other bean products(liang)																				
J21. Green vegetable/ brassica chinensis/bean seedling, etc.(liang)																				
J22. Cabbage, Chinese cabbage, cauliflower(liang)																				
J23. Celery(liang)																				
J24. Kidney bean/ long bean/cowpea(liang)																				

J25. Carrot(liang)																				
J26. Tomato(liang)																				
J27. Kidney bean/ long bean/cowpea(liang)																				
J28. Potato(liang)																				
J29. Champignon\ mushroom\ shiitake\oyster mushroom(laing)																				
J30. Pepper(liang)																				
J31. Bamboo shoot(liang)																				
J32. Agaric(liang)																				
J33. Seaweed, kelp(liang)																				
J34. Garlic(liang)																				

J35. Pickle (liang)																			
J36. Dry fruit (liang)																			
J37. Fruits (liang)																			

Part XII Pregnancy Related Disease History and Medical Records

1. Usually, how many people in your family have been eating meals together?

2. Since this pregnancy, how many meals did you eat at home per day?

During the 1st trimester ______times/day

During the 2nd trimester_____ times/day

During the 3rd trimester ______times/day

3. How much salt did your family eat per month? (unit: g) ______g

4. Was your mother (or sisters) informed that she once had Gestational hypertension diseases

0——No *1——Yes*

5. Is this pregnancy your first time of pregnancy (history of pregnancy include all pregnancies, like ectopic pregnancy, abortion, induced labour, etc.)

0——No *1——Yes*

6. If not the first time pregnancy, were you diagnosed as Gestational hypertension in the past pregnancies (record detailed diagnosis as much as possible, could be multiple records.)

0——No

1—— Yes

Diagnosis 1(Pregnancy outcome (gestational week, birth weight, gender, live birth or not) :______________

Diagnosis 2

Pregnancy outcome (gestational week, birth weight, gender, live birth or not)):_____________________

7. Do you have family history of chronic diseases, like hypertension, diabetes or cardiovascular diseases

0—— No
1—— Yes

Disease name ____ Relationship with you_________

8. *Was your mother (sisters) informed that she once had intrahepatic cholestasis of pregnancy (ICP)*
   0—— No *1——Yes*

9. If this pregnancy is not the first time, were you diagnosed as intrahepatic cholestasis of pregnancy (ICP) in the past pregnancies (record detailed diagnosis as much as possible, could be multiple records.)

0——No
1——Yes

Pregnancy outcome (gestational week, birth weight, gender, live birth or not)

Pregnancy outcome (gestational week, birth weight, gender, live birth or not)

10. *If you have delivery history, were your children diagnosed with following conditions (multiple choises)*
    1——Low birth weight infant *2——Macrosomia*
    3——Premature infant *4——Birth defect*
    5—Other defect (please indicate)

11. *Have you ever had induced labours because of the diagnosis of conception with defected fetus?*
    0——No
    1——Yes, the diagnosis is

12. *Since this pregnancy, did doctor tell you that you have Gestational hypertension diseases?*
    0—— No
    1——Yes

13. Gestational week when first diagnosed with gestational hypertention diseases

____

14． Detailed name of the diagnosed disease

____

15. Basal level of blood pressure ____________mmHg

16. Time of initial increase of blood pressure found Blood pressure ____mmHg

17. Maximum blood pressure during whole pregnancy Blood pressure ______mmHg

18. Blood pressure before medicine therapy Blood pressure ______mmHg

19. Blood pressure after medicine therapy Blood pressure ______mmHg

20. Blood pressure after delivery Blood pressure

21. Maximum level of blood pressure during natural delivery

Maximum level of blood pressure during caesarean section

22. Blood pressure after delivery

23. Does patient have any complications? _
0——No 1——Yes, diagnosis

24. Changes in the fundus oculi

25． Since this pregnancy, were you diagnosed with intrahepatic cholestasis
0——No 1——Yes

26. Gestational week when first diagnosed ________
27. Initial cholic acid level

28. Maximum cholic acid level during the entire pregnancy____

29. Cholic acid level before medicine therapy____

30. Cholic acid level after medicine therapy____

31. Names of the medicines____ Dose____

Total days of using medicines____

32. Cholic acid level after delivery____
